# Microbial abundance on the eggs of a passerine bird and related fitness consequences between urban and rural habitats

**DOI:** 10.1371/journal.pone.0185411

**Published:** 2017-09-27

**Authors:** Sang-im Lee, Hyunna Lee, Piotr G. Jablonski, Jae Chun Choe, Magne Husby

**Affiliations:** 1 School of Undergraduate Studies, Daegu Gyeongbuk Institute of Science and Technology, Daegu, Korea; 2 Laboratory of Behavior and Ecology, EcoCreative Program, Ewha Womans University, Seoul, Korea; 3 Laboratory of Behavioral Ecology and Evolution, School of Biological Sciences, Seoul National University, Seoul, Korea; 4 Museum and Institute of Zoology, Polish Academy of Sciences, Warsaw, Poland; 5 Department of Science, Nord University, Levanger, Norway; Charles University, CZECH REPUBLIC

## Abstract

Urban environments present novel and challenging habitats to wildlife. In addition to well-known difference in abiotic factors between rural and urban environments, the biotic environment, including microbial fauna, may also differ significantly. In this study, we aimed to compare the change in microbial abundance on eggshells during incubation between urban and rural populations of a passerine bird, the Eurasian Magpie (*Pica pica*), and examine the consequences of any differences in microbial abundances in terms of hatching success and nestling survival. Using real-time PCR, we quantified the abundances of total bacteria, *Escherichia coli/Shigella* spp., surfactin-producing *Bacillus* spp. and *Candida albicans* on the eggshells of magpies. We found that urban magpie eggs harboured greater abundances of *E*. *coli/Shigella* spp. and *C*. *albicans* before incubation than rural magpie eggs. During incubation, there was an increase in the total bacterial load, but a decrease in *C*. *albicans* on urban eggs relative to rural eggs. Rural eggs showed a greater increase in *E*. *coli/Shigella* spp. relative to their urban counterpart. Hatching success of the brood was generally lower in urban than rural population. Nestling survival was differentially related with the eggshell microbial abundance between urban and rural populations, which was speculated to be the result of the difference in the strength of the interaction among the microbes. This is the first demonstration that avian clutches in urban and rural populations differ in eggshell microbial abundance, which can be further related to the difference in hatching success and nestling survival in these two types of environments. We suggest that future studies on the eggshell microbes should investigate the interaction among the microbes, because the incubation and/or environmental factors such as urbanization or climate condition can influence the dynamic interactions among the microbes on the eggshells which can further determine the breeding success of the parents.

## Introduction

Urban environments present novel habitats to many bird species [1]. Urban areas are different from rural areas in many aspects; urban environments are generally warmer [2], more polluted (in terms of chemicals, e.g. [3]), and have different light conditions [4] than adjacent rural environments. These environmental conditions may be challenging for birds that are new to urban areas. A higher prevalence of some diseases in urban areas has been described [5, 6], which might be related to human activities such as feeding [6] or low diversity of the commensals that can protect the hosts against the pathogens [7], but so far there is no general consensus on the prevalence of parasites and pathogens between rural and urban environments (e.g. some ticks are more common in natural areas [8–10]). Microbial community in the urban environment has recently been studied in soil, and these studies suggested that urban environment contains different composition of microbes from rural environment [11–13]. However, it remains unclear how these differences in microbial communities between urban and rural environment would affect wildlife living in each environment.

Environmental microbes can affect the wildlife in a variety of ways. For birds, environmental microbes can be transmitted to the eggshells via nest materials or parental mediation. Recent studies have associated microbial flora on the surface of eggs with hatching success [14–16]. If urban and rural areas differ in their microbial flora, one can predict that there should also be a difference in the microbial flora in nests and on eggshells of resident birds. In spite of the recent developments of molecular tools that enable detailed analyses of microbial communities, how urban environments shape microbial communities and further influence hatching and breeding success of birds has never been studied. For example, very little is known about the effects of urban versus rural habitats on the abundance of bacteria in the clutches of typically rural birds that have recently settled in urban habitats.

To better understand the dynamics of urban versus rural microbial communities and how they affect fitness, we compared the two types of habitats in terms of abundance of total bacteria and chosen representatives of microbes in the clutches of the Eurasian Magpie (*Pica pica*). Specifically, we compared microbial abundance and change in relative abundance on magpie eggshells in urban and rural areas over three weeks of incubation. Microbial abundance on the surface of eggs is important for survival of the embryos. Bacterial infections can occur through the eggshell and decrease egg viability [15, 17–19] or the hatchling phenotype [20]. Incubation is known to decrease the probability of such bacterial infections by drying the egg surface and thus to supress microbial proliferation in general [21–23] or to suppress the growth of bacteria that are vulnerable to dehydration [24, 25]. Apart from the effect of incubation behaviour on microbial abundance on eggshells, eggshell microbial community can be determined by microbes of maternal origin (such as those transmitted during the egg-laying through cloacal contact [23, 26, 27] or from the feathers/skin of the mother during incubation [28]), and those of environmental origin that both parents and eggs are exposed to (such as from nest materials [29–31]).

We asked three primary questions; (i) whether birds that breed in urban areas have different microbial abundance on the eggshells than those that breed in rural areas, (ii) whether the microbial abundance change due to incubation is different in urban versus rural areas, and (iii) whether any difference in breeding success between rural and urban areas can be attributed to the difference in microbial abundance. In order to address these questions, we chose to examine the abundance of microbes on the eggshells of the Eurasian Magpie. Eurasian Magpies are widely distributed throughout the Northern Hemisphere and have successfully adapted to the urban environments [32, 33]. Thus, they are optimal species with which to study differences in microbial abundance between urban and rural environments.

## Materials and methods

### Ethics statement

The research has been conducted according to relevant national and international guidelines. The procedure for this study has been approved by Institutional Animal Care and Use Committee of Seoul National University (No. SNU 130621–6). All landowners received written information about the project, and gave permission to use the nests in this investigation.

### Sampling

Microbial samples were collected from magpie eggs in urban (central Trondheim city, 63° 25' N, 10° 25' E; N = 17 nests) and rural (Skatval, 63° 30' N, 10° 48' E; N = 14 nests) areas in Norway. The closest distance between magpie nests in the two areas was 19.7 km, and the two sites were separated by a fiord. The urban sites consisted primarily of private residences with gardens and paved roads. The rural area consisted of mostly cultivated farmland with 50–320 m between each farm, but also contained patches of forests. The investigated urban area was about 0.9 km^2^, and the rural area was about 25.8 km^2^. Magpies are normally sedentary [32, 34], and are especially reluctant to cross open water [35]. Thus it is not likely that the magpies in the two areas in this study shared nest materials.

Nests were checked twice a week during the egg laying period. If the laying date of the first egg was missed, we estimated the laying date assuming that one egg was laid per day [32]. Tree height and nest height were measured by the use of Suunto trigonometric altimeter. Except for clutch size being larger in rural population, characteristics of the nests and laying dates did not differ between the two sites (S2 Table).

In magpies, full incubation usually begins after three or four eggs have been laid and the chicks hatch around 22 days after the first egg is laid [32, 36]. Thus, we sampled microbes from the eggshells at the early incubation stage (usually when the nest contained three eggs; hence noted as “day 3”) and at the late incubation stage (usually 18 days after the first egg-laying; hence noted as “day 18”). Before sampling, researchers wore plastic gloves sterilized with 96% ethanol. One egg was randomly selected in each nest at the early incubation stage and another egg was randomly selected at the late incubation stage. The first egg was marked with non-toxic permanent marker, and never included in the late incubation sampling. Microbes on the eggshell were sampled by thoroughly rubbing the whole eggshell surface with a sterile cotton swab treated with sterile 1× PBS buffer (pH 7.4) [37]. As the sampled eggs differed in size, the length and width of the sampled eggs were measured using digital callipers at day 18 after microbial sampling, and we estimated surface area using the following equation [38];

$$S=3\times L^{0.771}\times W^{1.229}$$

where S, L and W are the surface area, length and width of the egg, respectively. After sampling, swabs were placed in a 1.5ml tube containing 500 μL of 1× PBS buffer, and these samples were kept in an icebox temporarily for 3–4 hrs before they were stored at -50°C until DNA extraction and PCRs were conducted.

Shortly after hatching (< 5 days after hatching), we accessed to the nests in order to determine the number of hatchlings, and we repeated this again at day 18 to assess the number of surviving hatchlings. These values were used in the analysis of hatching success and nestling survival respectively (see *Statistical analyses*).

### Preparation of standards for qPCR

Three target microbial taxa were chosen based on previous pyrosequencing results [24] and the results of preliminary screening: *Escherichia coli/Shigella* spp., surfactin-producing *Bacillus* spp. and *Candida albicans*. For estimating *E*. *coli*/*Shigella* spp., we used UAL1939b and UAL2015b primers. Although this primer set is widely used for estimating *E*. *coli* abundance (e.g. [39–42]), this primer set cannot discriminate *E*. *coli* from *Shigella* spp. *Escherichia coli* includes a variety of strains that are both pathogenic and non-pathogenic. Pathogenic *E*. *coli* is known to cause intestinal diseases [43, 44], which would be detrimental to the embryos [45, 46], but is also found in apparently healthy birds [47]. On the other hand, *Shigella* is not normally cultured from birds [48] and its effect on embryos of wild birds remains unknown. Since it was suggested that *E*. *coli* and *Shigella* species belong to the same species and the distinction was maintained for clinical and practical, not biological, reasons [49, 50], differentiating *E*. *coli* from *Shigella* might not be biologically meaningful. Thus, in this study, we do not distinguish the two microbes and we refer to the taxonomic group that is detected by the primer set as *E*. *coli*/Shigella spp.

Surfactin is a powerful antibiotic lipopeptide that can disintegrate cell membranes [51, 52] and some species of *Bacillus* have surfactin gene (*sfp*) that is responsible for surfactin production [53, 54]. Among them, *B*. *subtilis* and *B*. *licheniformis* are commonly present in soils and the feathers [55–57]. The presence of surfactin-producing *Bacillus* on the eggshells potentially increases survival of embryos as surfactin can deter the proliferation of other potentially pathogenic microbes [24, 28, 30, 31, 58].

*Candida albicans* is an opportunistic fungal pathogen that is known to cause a variety of diseases including infections in mucous membranes and urogenital tissues in birds [59]. In particular, *C*. *albicans* can cause serious infections that can be lethal in immune-suppressed or compromised individuals including developing embryos [60, 61]. As *C*. *albicans* is commonly found in the environment and the immune systems of the embryos inside the eggs that are still developing, a greater *C*. *albicans* load on the eggshell potentially leads to greater egg mortalities [61].

In order to quantify the abundance of these microbes, we conducted quantitative PCRs. For the quantification of total bacterial load, we used the protocol of our previous study [24]. For *C*. *albicans*, we obtained the type culture from KCTC (Korean Collection for Type Cultures; type No. KCTC 27241). The other microbes were donated by Dr. Chun Jong Shik’s laboratory in Seoul National University (type Nos. JC 2021 for *E*. *coli*, and JC 2885 for *B*. *subtilis* as a representative of surfactin-producing *Bacillus*). We extracted DNA from the type cultures using the MO BIO PowerSoil^®^ DNA Isolation kit following manufacturer’s protocol (MO BIO Laboratories, Carlsbad, CA, USA). The only modification was that both buffer and the head of the swab was placed in the PowerBead Tubes. Extracted DNA was amplified (FastMix/French^™^ PCR, Intron, Seongnam, Korea) with respective primers (Table 1). PCR products were inserted into the cloning vectors (pGEM^®^-T Easy Vector Systems, Promega, Madison, WI, USA) which were transformed in competent cells (Hit-DH5α, RBC Bioscience, Taipei, Taiwan). Recombinant plasmids were selected on LB (Luria-Bertani) plates with ampicillin (100μg/ml), IPTG (isopropyl β-D-thiogalactoside, 0.5mM), and X-Gal (5-bromo-4-chloro-3-indolyl β-d-galactoside, 80μg/ml). A single recombinant colony was picked and grown in LB in a shaking incubator at 30°C or 37°C overnight. The recombinant plasmid DNA was then purified by using a plasmid mini prep kit (Hybrid-Q^™^Plasmid Rapidprep, GeneAll Biotechnology, Seoul, Korea). The quality and quantity of plasmid DNA were estimated with a NanoDrop ND-1000 Spectrophotometer (NanoDrop Technologies Inc., Wilmington, DE, USA) and a TBS-380 Mini-Fluorometer (Turner Biosystems, Sunnyvale, CA, USA) with Quant-iT^™^ picogreen^®^ dsDNA assay kit (Invitrogen, Carlsbad, CA, USA), respectively. The amount of DNA molecules was used to estimate the DNA copy number using the following formula [62]:

$$\text{DNA}\;\text{copy}\;\text{number}=\frac{6.02\times 10^{23}(copy/mol)\times DNA\;amount\;(g)}{DNA\;length\;(bp)\times 660\;(g/mol/bp)}.$$

**Table 1:** Microbes and primer sequences. Target microbes and the primer sequences used in this study.

Target microbe	Primer sequences	Source
Total bacteria	338F: 5’-ACT CCT ACG GGA GGC AGC AG-3’ 518R: 5’-ATT ACC GCG GCT GCT GG-3’	[83]
*Escherichia coli/Shigella* spp.	UAL1939b: 5’-ATG GAA TTT CGC CGA TTT TGC-3’ UAL2105b: 5’-ATT GTT TGC CTC CCT GCT GC-3’	[84]
Surfactin-producing *Bacillus* spp.	*sfp*-f: 5’-ATG AAG ATT TAC GGA ATT TA-3’ *sfp*-r: 5’-TTA TAA AAG CTC TTC GTA CG-3’	[53]
*Candida albicans*	CA: 5’-ATT GCT TGC GGC GGT AAC GTC C-3’ CTSR: 5’-TCT TTT CCT CCG CTT ATT GAT ATG C-3’	[85, 86]

### Quantitative PCR

After extracting DNA from the swab samples, we amplified the DNA from the target microbial taxa with a Rotor-Gene^®^ Q (QIAGEN, Venlo, Limburg, Netherlands) real-time PCR system. Real-time PCR assays were conducted with 5 μL of 2× Rotor-Gene SYBR Green PCR Master Mix, 3.5 μL of RNase-Free Water (Rotor-Gene SYBR Green PCR Kit, QIAGEN, Venlo, Limburg, Netherlands), 0.25 μL of 10 pmol/L for forward and reverse primers (Table 1), and 1 μL of DNA extract in the reaction volume of 10 μL. Thermal condition for PCR was as follows; initial activation for 10 min at 95°C, PCR cycling for 10 sec at 95°C and for 30 sec at 52°C (for surfactin-producing *Bacillus* spp.) or 55°C (for *C*. *albicans*) or 60°C (for total bacteria and *E*. *coli/Shigella* spp.) for 40 times, and melting curve were obtained by lowering the temperature from 95°C to 55°C (descending by 1°C each step). In order to have a reliable estimate for microbial abundance, PCR was conducted at least three times for each sample and the average value was calculated and used as the data for the statistical analysis. Since it is generally known that the melting curves are unstable when the primer-dimers are present, we manually checked the shape of melting curves. Threshold cycle (Ct) values of the samples were converted to DNA copy numbers with Rotor-Gene^®^ Q software. Standards were serially 10-fold diluted from 1×10^0^ to 1×10^8^ copies/μL and the efficiency of the standards were between 90% and 105%, and R^2^ was higher than 0.97. The calculation for estimating the microbial abundance involves converting copy numbers from the Ct values that were obtained with Rotor-Gene^®^ Q software. For instance, copy numbers for *Candida albicans* varied from 0.008 (nest 17) to 6.293 (nest 34), and dividing these values with the respective area of egg surface that was sampled (18.962 cm^2^ and 25.598 cm^2^) gives us the values of 0.0004 and 0.2458 respectively. After adding 1 to these values and taking logarithm with the base of 10 gives us the values of 0.0002 and 0.0955 respectively.

### Climate variables

Since the difference in weather conditions can shape microbial abundance in urban and rural population, we conducted additional analyses on the relationship between climate variables and microbial abundance. Daily average ambient temperature and relative humidity data were collected from the closest official climate station at Værnes (63.46°N and 10.94°E). For each nest we calculated the average temperature and humidity in early (day 0–3) and late incubation (day 15–18), and the difference in temperature between these two stages (i.e. day 15–18 temperature subtracted by day 0–3 temperature).

### Statistical analyses

#### Microbial abundance

The microbial abundance, estimated as copy number of microbes per cm^2^ of eggshell, was log-transformed before the statistical analyses (since there were “0” values in the abundance data of *C*. *albicans*, we first added “1” to the abundance values and conducted the log-transformation). We adopted two statistical approaches to understand the variations in the microbial abundance data. Firstly, we used an aligned rank test [63, 64] to compare the microbial loads on the eggshell surfaces between urban and rural areas at day 3 and at day 18 separately. Aligned rank tests can handle non-normal, left-censored data with outliers that are characteristic of microbial abundance data [65]. We calculated aligned ranks using ARTool [66, 67] and conducted one-way ANOVA using PROC MIXED in SAS version 9.4 (SAS Institute, Cary, NC). We conducted statistical comparisons separately on the data from both day 3 and day 18.

If we use the rank-based analyses only, the information that can be obtained from the paired responses between day 3 and day 18 would be lost. The rank-based analysis would be appropriate for a cross-sectional comparison, but it would not reveal any changes in microbial loads that occurred during incubation. Thus, we added a Wilcoxon signed-rank test to compare the changes in microbial loads on the magpie eggs in urban and in rural areas. Change in microbial load was calculated as the difference between load on day 18 and day 3. For this test, we used PROC UNIVARIATE in SAS version 9.4.

#### Factors for the microbial abundance

To characterize microbial abundance and use the values for further analyses, we extracted principal components (PCs) from the microbial abundance data and used up to three PCs in the statistical analyses. We used PCs instead of raw microbial abundance data, because some of the microbial abundances were correlated (S3 Table) and we had relatively small sample sizes with which to run the statistical model with many variables. PC extraction was conducted from three separate datasets: (1) day 3 abundance (5 variables), (2) day 18 abundance (5 variables), and (3) day 3 abundance and the difference between day 3 and day 18 abundances (hence “Δ abundance”) (10 variables). PC extraction was conducted with PROC PRINCOMP in SAS. As the first three PCs explained > 80% of the variance in the data (S4 Table), we included them as the response variables representing microbial abundance and sought for relationship with average temperature, average humidity, and population. Climate factors were calculated to reflect the weather condition that may affect the abundance of eggshell microbes; average temperature and humidity between days 0–3 and days 15–18 were used in the models that concern PCs extracted from day 3 microbial abundance and day 18 abundance respectively; in the model whose response variable was PCs extracted from day 3 and Δ abundance, we included average temperature and humidity between days 0–3, average temperature difference between days 15–18 and 0–3, and average humidity between days 15–18. We first considered the interaction between population and climate variables but none of these interaction terms were significant so we present the result with the main effects only.

#### Hatching success and nestling survival

Difference in hatching success and nestling survival between rural and urban areas was examined using logistic regression (PROC GENMOD in SAS) with PCs for microbial abundance as the explanatory variables. We allowed interaction terms between the population and PCs and conducted backward elimination procedure [68]. We treat each nest as an independent unit, as magpies are strongly territorial during the breeding season [32]. The raw data is provided in S1 Table.

## Results

### Comparison between rural and urban populations

In general, changes in the microbial abundance during the incubation (Δ abundance) were negatively correlated with the initial abundance (S3 Table).

**Fig 1:**
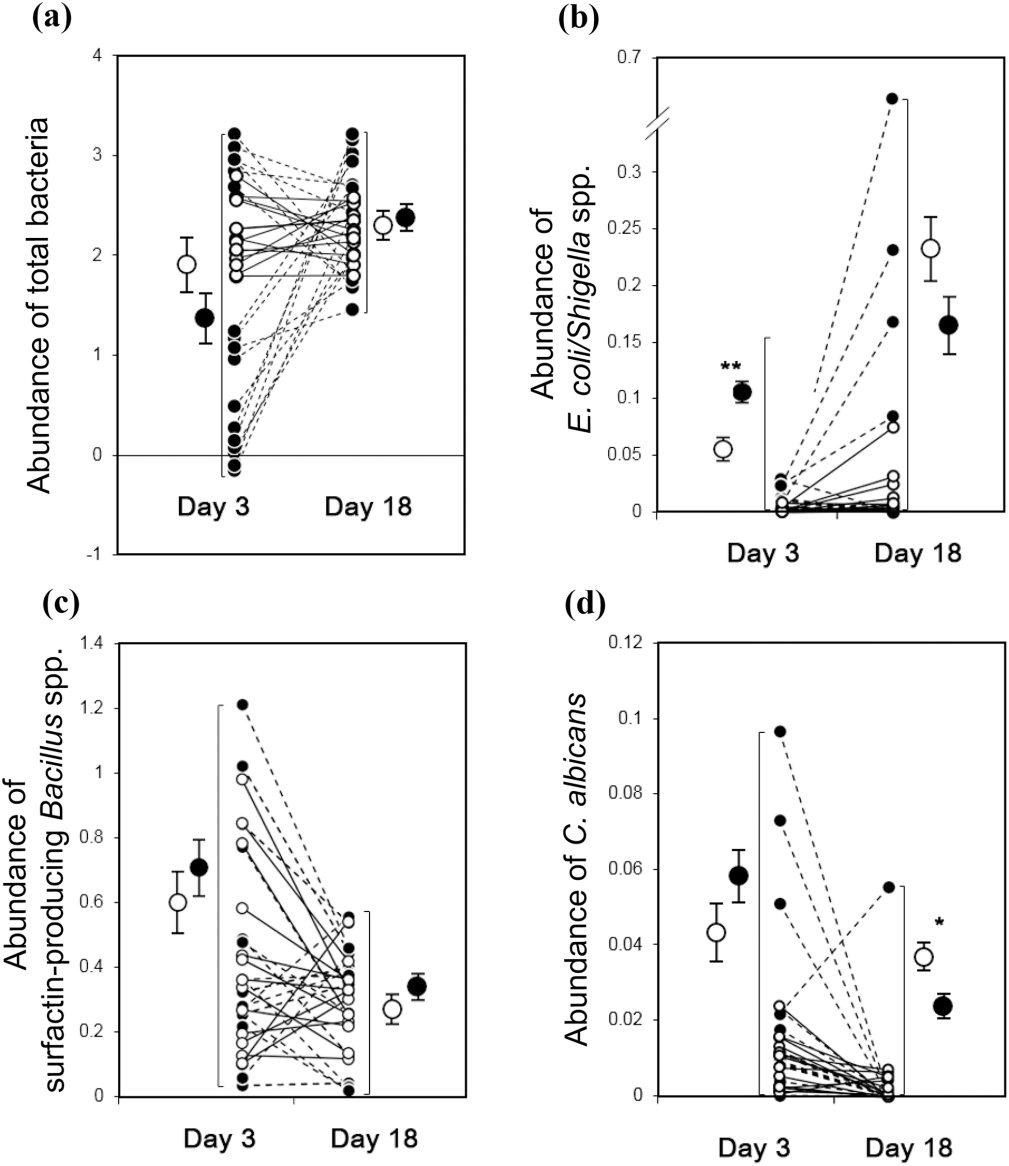
Changes in microbial abundance. Comparisons of changes in the microbial abundance on the naturally incubated magpie eggs between rural (n = 14 nests) and urban (n = 17 nests) populations; (a) total bacteria, (b) *Escherichia coli/Shigella* spp., (c) surfactin-producing *Bacillus* spp., and (d) *Candida albicans*. Paired responses of each nest (connected circles) are given along with the average aligned ranks (circles with error bars). Open circles (connected with solid lines) and closed circles (connected with dashed lines) denote the responses of the rural and urban populations respectively. Units of Y axis values for the microbial abundance are log_10_ (copy numbers of microbes) per cm^2^ of the eggshells for (a), (b) and (c), and log_10_ (copy numbers+1) per cm^2^ of the eggshells for (d). Error bars for the aligned ranks denote standard errors. Significance level of the comparison of aligned ranks is given as “*” for 0.01<P<0.05 and “**” for 0.001<P<0.01.

Results from the aligned rank tests suggest that there was no significant difference in total bacterial load between urban and rural areas, either at day 3 or at day 18 (circles with error bars in Fig 1a, Table 2). However, signed rank tests suggest that total bacterial loads on the magpie eggs in the urban area increased while those in the rural area did not change much (circles connected with lines in Fig 1a, Table 3).

**Table 2:** Microbial abundance in urban and rural clutches of magpies at day 3 and day 18.

Stage	Microbial entity	F value	Summary
Day 3	Total bacteria	2.47	
	*Escherichia coli/Shigella* spp.	3.73^0.06^	Urban ≥ Rural
	Surfactin-producing *Bacillus* spp.	0.29	
	*Candida albicans*	1.09	
Day 18	Total bacteria	0.47	
	*Escherichia coli/Shigella* spp.	1.84	
	Surfactin-producing *Bacillus* spp.	0.85	
	*Candida albicans*	14.09***	Urban << Rural

Results of aligned rank tests on the microbial abundance on magpie eggshells. Numerical and denominator degrees of freedom were 1 and 36 respectively.

Statistical significance is noted as “*” for 0.01<P<0.05 and “***” for P<0.0001.

P value of one marginally non-significant result is shown as a superscript.

Raw data is provided in S1 Table.

**Table 3:** Changes in microbial abundance. Results of Wilcoxon signed-rank tests on the changes in microbial loads. The change in microbial loads was calculated by subtracting microbial load at day 3 from that at day 18; thus positive signed-rank value mean increases in microbial loads during incubation and vice versa.

Microbial entity	Naturally incubated magpie eggs (n = 31)			
	Rural (n = 14)		Urban (n = 17)	
	S	P	S	P
Total bacteria	6.5	0.715	47.5	0.023
*Escherichia coli/Shigella* spp.	40.5	0.009	-6.5	0.782
Surfactin-producing *Bacillus* spp.	-23.5	0.153	-31.5	0.145
*Candida albicans*	-42.5	0.005	-62.5	0.002

At day 3, the magpie eggs in the urban population harboured nearly significantly more *E*. *coli/Shigella* spp. than rural magpie eggs (Fig 1b, Table 2). However, unlike in the urban magpie nests, *E*. *coli/Shigella* spp. increased on the surface of rural magpie eggs in the majority of nests (Table 3). Therefore, the initially nearly significant difference between these two populations became strongly non-significant at day 18 (Fig 1b, Table 2).

There was no significant difference in the abundance of surfactin-producing *Bacillus* spp. between urban and rural magpie eggs neither at day 3 nor at day 18 (Fig 1c, Table 2). Furthermore, there was no statistically significant change in the abundance of surfactin-producing *Bacillus* spp. with incubation (Table 3).

The abundance of *C*. *albicans* decreased with incubation time (Fig 1d; Table 3). The abundance of *C*. *albicans* did not differ between urban and rural areas at day 3, but at day 18 rural magpie eggs harboured more *C*. *albicans* than urban magpie eggs (Fig 1d; Table 2), implying that the decrease in *C*. *albicans* load was less pronounced in rural magpie eggs.

### Factors for the microbial abundance

We extracted three PCs from the microbial abundance data and investigated the effects of temperature, humidity, and population (the result is given in S5 Table). The interactions between population and climate variables were first considered but not significant and thus removed. The climate conditions did not differ between urban and rural populations (*t*-test, all P>0.14). Among the PCs, PC1 and PC3 from day 3 abundance were related with average temperature during day 0 and 3, and PC2 and PC3 from day 18 abundance were related with average humidity during day 15 and 18. All PCs from day 3 and Δ abundances were related with average temperature during day 0 and day 3. Specifically, PC1 from day 3 abundance was greater in the urban population and was also positively related to average temperature. PC1 from day 3 and Δ abundances were also greater in the urban population. These two PCs were positively loaded with the abundances of *C*. *albicans* and *E*. *coli/Shigella* spp. (S4 Table).

### Hatching success and nestling survival

**Fig 2:**
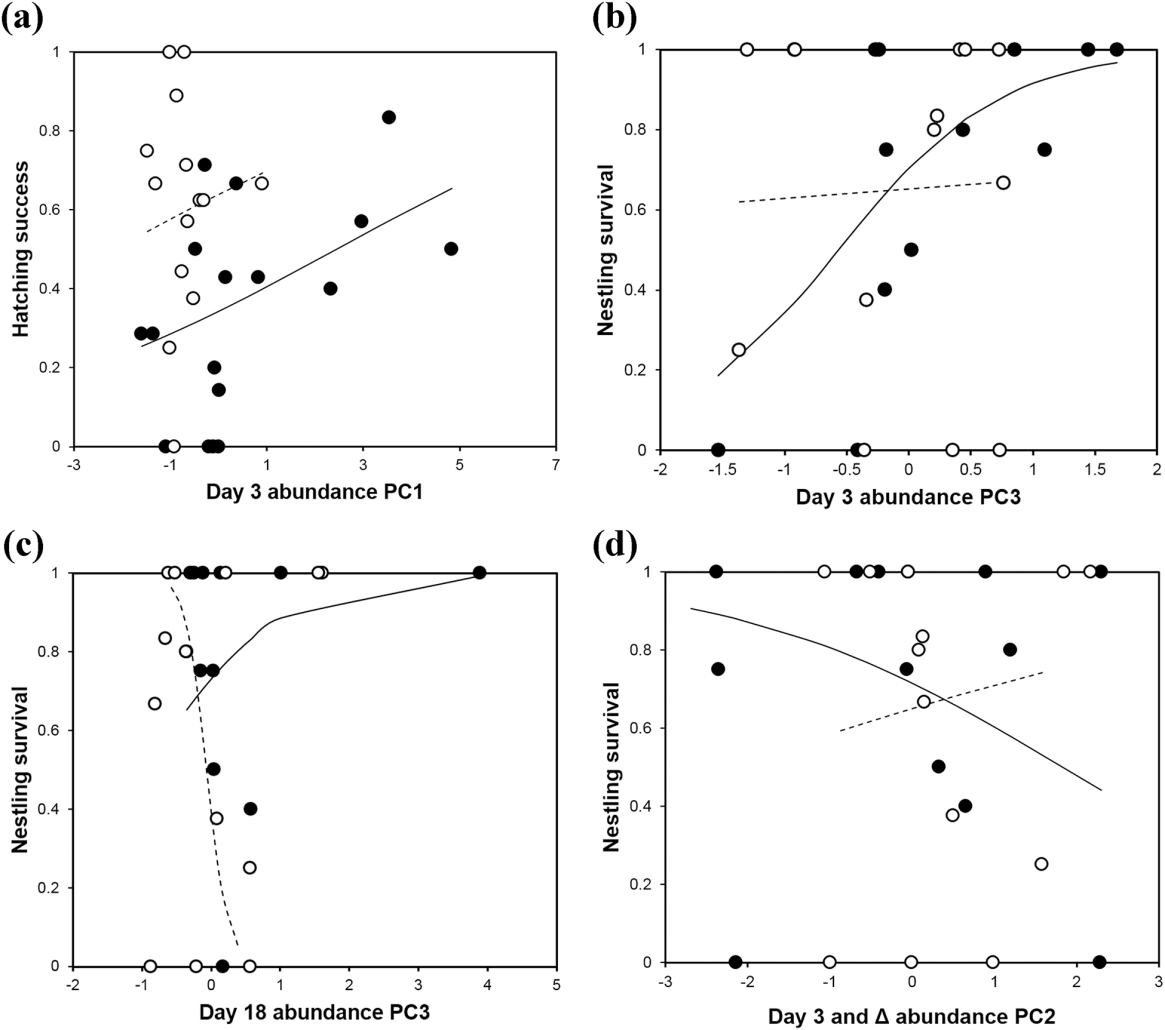
Microbial abundance, hatching success and nestling survival. Relationship between microbial abundance and hatching success (a) and nestling survival (b)–(d). Open circles (fitted values marked with dashed lines) and closed circles (fitted values marked with solid lines) denote the responses of the rural and urban areas respectively. For microbial abundance, principal components (PCs) extracted from day 3 abundance, day 18 abundance, or day 3 and Δ abundance were used (see *Statistical analyses*).

Hatching success of the egg was significantly lower in the urban than the rural population (Table 4). In addition to this general difference, microbial abundance was related with hatching success: PC1 from day 3 abundance and PC1 extracted from day 3 and Δ abundances were positively related with the hatching success of broods (Fig 2a for the former; for the latter the pattern was similar). These PCs were both positively loaded with *C*. *albicans* and *E*. *coli/Shigella* spp. abundance at day 3 (S4 Table). Thus, these results suggest that the abundances of *C*. *albicans* and *E*. *coli/Shigella* spp. at day 3 are positively related with the hatching success of broods. Insignificant interactions between PCs and populations were removed from the model.

**Table 4:** Microbial abundance, hatching success and nestling survival. The relationships between population and microbial abundance on the hatching success and the nestling survival.

Response variable	Stage considered for PC extraction	Variable	Estimate±SE	df	χ^2^	P
Hatching success	Day 3	Population	Rural: 0.577±0.330	1	14.62	0.000
			Urban: -0.648±0.225			
		PC1	0.266±0.113	1	5.75	0.017
	Day 18	Population	Rural: 0.392±0.287	1	9.42	0.002
			Urban: -0.480±0.204			
	Day 3 and Δ	Population	Rural: 0.536±0.325	1	12.84	0.000
			Urban: -0.600±0.218			
		PC1	0.174±0.092	1	3.51	0.061
Nestling Survival	Day 3	Population		1	0.24	0.624
		PC3		1	6.38	0.012
		Population × PC3	Rural: 0.099±0.711	1	4.89	0.027
			Urban: 1.524±0.605			
	Day 18	Population	Rural: -0.764 ±0.689	1	10.40	0.001
			Urban: 1.219±0.427			
		PC3		1	12.74	0.000
		Population × PC3	Rural: -7.345±2.278	1	25.60	0.000
			Urban: 0.638±0.559			
		PC2	-0.623±0.275	1	6.12	0.013
	Day 3 and Δ	Population		1	1.47	0.226
		PC2		1	0.21	0.649
		Population × PC2	Rural: 0.607±0.474	1	5.23	0.022
			Urban: -0.399±0.274			
		PC3	-0.607±0.217	1	8.21	0.004

In contrast, microbial abundance was differentially related with survival of nestlings between rural and urban populations (Table 4, Fig 2); lower initial abundance of surfactin-producing *Bacillus* spp. and higher initial abundance of *C*. *albicans* on eggshells (PC3 from day 3 abundance; S4 Table) was positively related to nestling survival only in the urban population (Fig 2b). Lower abundance of *C*. *albicans* and higher abundance of *E*. *coli/Shigella* spp. near hatching (PC3 from day 18 abundance; S4 Table) was positively related with nestling survival in the urban population but negatively so in the rural population (Fig 2c). Lower initial abundance of total bacteria and greater increase in total bacterial load and the abundance of surfactin-producing *Bacillus* spp. (PC2 from day 3 and Δ abundance) was positively related with nestling survival in the urban population but negatively so in the rural population (Fig 2d).

## Discussion

Our results show for the first time that urban and rural populations differ in the initial abundance of microbes on the eggs of wild birds and in the changes in composition of microbial flora during incubation. We found that magpie eggs in the urban population harboured greater abundance of *E*. *coli/Shigella* spp. before incubation than eggs in the rural population. During incubation, there was a greater increase in the abundance of total bacteria but a greater decrease in the abundance of *C*. *albicans* on urban magpie eggs, whereas *E*. *coli/Shigella* spp. abundance increased on rural magpie eggs. Considering that incubation keeps the surface of the eggshell warm and dry [22, 25], and many microbes, including those that were examined in our study, show optimal growth in warmer and humid conditions [17, 69–71], the fact that the abundance of *E*. *coli/Shigella* spp. increased whereas that of surfactin-producing *Bacillus* spp. and *C*. *albicans* decreased during the incubation suggests three, not exclusive, possibilities; (i) the interaction between the microbes shaped the changes in the microbial abundance on the eggshell; (ii) eggshell surfaces are exposed more or longer to some microbes than the others (e.g. some *Bacillus* that are present in the feathers may have more chances to be transmitted to eggshell from the feathers of incubating mothers than other microbes); and/or (iii) the conditions that are optimal for the growth of these microbes differ slightly. Before the onset of full incubation, the abundance of the three types of microbes was positively related with the ambient temperature, but during the incubation this relationship disappears except surfactin-producing *Bacillus* spp.; change in average temperature during incubation was positively related with the change in the abundance of total bacteria and surfactin-producing *Bacillus* spp. Based on the minimum water activity level for the growth of the microbes, we can expect that *Bacillus* (*B*. *subtilis*, as the representatives of surfactin-producing *Bacillus* spp., *a*_*w*_ = 0.91) and *C*. *albicans* (*a*_*w*_ = 0.88) may grow better on incubated (thus dried) eggshells than *E*. *coli* (*a*_*w*_ = 0.95) [72]. However, what we observed is that both surfactin-producing *Bacillus* spp. and *C*. *albicans* decreased whereas *E*. *coli/Shigella* spp. increased during incubation. The discrepancy between this expectation and what was observed indicates that the third possibility cannot be solely responsible for the changes in microbial abundance on magpie eggshells during incubation.

Despite the greater reduction in *C*. *albicans* and the lower increase in *E*. *coli/Shigella* spp. on urban magpie eggs during the incubation, hatching success of the urban magpie eggs was generally lower than that of the rural magpie eggs. This suggests that *C*. *albicans* and *E*. *coli/Shigella* spp. might not negatively affect hatching success at the level of abundance found in our study. Currently there is no real-time PCR-based estimation on the abundance of *C*. *albicans* and *E*. *coli/Shigella* spp. that cause embryo mortality in wild birds. Considering that *E*. *coli* abundance ranges from 5–6 log_10_ copies/g in healthy chicken guts [73], and ~1.87 log_10_ copies/g in healthy chicken faeces [74], the abundance of *E*. *coli/Shigella* spp. detected in our study (~0.6 log_10_ copies/cm^2^) seems very low, although a direct comparison is difficult due to the difference in the units. A small increase in *E*. *coli/Shigella* spp. or *C*. *albicans* abundance may not be harmful, but rather beneficial to hatching success, as these organisms can directly or indirectly inhibit the growth of more pathogenic microbes. Indeed, we found that the initial abundance of *C*. *albicans* and the associated abundance of *E*. *coli/Shigella* spp. (PC1 from day 3 abundance) was positively related to hatching success, and this effect was independent of the effect of population. Even though there are pathogenic strains of *E*. *coli*, some strains of *E*. *coli* produce bacteriocins [75]. Alternatively, small increase in the abundance of microbes may not necessarily impair but rather stimulate the immune system of embryos. Currently we cannot discern how this positive relationship between microbial abundance and hatching success is formed.

Overall (not population-specific) nestling survival was more related with the abundance of surfactin-producing *Bacillus* spp. and *C*. *albicans*, than that of *E*. *coli/Shigella* spp. or total bacterial load. Greater initial abundance of surfactin-producing *Bacillus* spp. and greater increase of *C*. *albicans* during incubation (PC3 of day 3 and Δ abundance) and thus the abundance of these two microbes near the end of the incubation (PC2 of day 18 abundance) were negatively related to nestling survival. This result implies that (i) eggshell microbes may have more long-term effects than previously recognized, and (ii) their short-term effects may be different from long-term effects.

In addition to this relationship, we found that microbial abundance on eggshells differentially relates with nestling survival in rural and urban populations. As far as we know, this is the first report on such case. In the urban population, nestling survival was greater with lower initial abundance and greater increase of surfactin-producing *Bacillus* spp. during incubation, lower total bacterial load and higher *C*. *albicans* abundance before incubation, and lower *C*. *albicans* abundance and higher *E*. *coli/Shigella* spp. abundance near hatching. In the rural population, nestling survival was greater with higher total bacterial load before incubation, lower increase in the abundance of surfactin-producing *Bacillus* spp. during incubation, and higher *C*. *albicans* abundance and lower *E*. *coli/Shigella* spp. abundance near hatching. In our data, not only the initial abundance of these three types of microbes were positively correlated with one another but also the initial abundance of one was related with the change in the abundance of the others (initial *E*. *coli/Shigella* spp. abundance was negatively related with the change in the abundance of surfactin-producing *Bacillus* spp. and *C*. *albicans*; initial *C*. *albicans* load was positively related with the change in the abundance of *E*. *coli/Shigella* spp.). Interactions between these microbes have been reported, albeit not specifically in birds. For instance, *C*. *albicans* and *E*. *coli* exhibit a cooperative interaction wherein *E*. *coli* enhances adhesion of *C*. *albicans* [76]. *Bacillus subtilis*, as the representative of surfactin-producing *Bacillus*, and *E*. *coli* can produce bacteriocins against each other [75], and co-existence with *E*. *coli* can promote sporulation of *B*. *subtilis* [77]. *Bacillus subtilis* can also show antifungal effects against *C*. *albicans* [78]. Thus, it is likely that the strength and dynamics of the interaction among the microbes on the magpie eggshells differ between urban versus rural environments, which might be responsible for the difference in the relationship between microbial abundance and nestling mortality in these two environments.

How the difference in microbial abundance on the magpie eggshell is formed between urban and rural environments remains to be studied. Among the three types of microbes that were studied in this study, greater increase in *E*. *coli/Shigella* spp. abundance on rural magpie eggs may have been the result of greater exposure of rural magpies to *E*. *coli* during foraging. In Norway, faecal waste from domestic animals is spread on farmland areas every spring as a natural fertilizer (M. Husby, pers. obs). Similarly, *C*. *albicans* and some surfactin-producing *Bacillus* are found in the soil [55, 56, 79]. Especially, some surfactin-producing *Bacillus* such as *B*. *subtilis* and *B*. *licheniformis* are known to be present in the feathers [56, 57], and they could be transferred to the eggshell from the feathers on the mother’s belly that directly make contact with eggshell. However, in our results, the abundance of surfactin-producing *Bacillus* spp. did not differ between urban and rural magpie eggs. Moreover, if mother’s plumage is an important source for surfactin-producing *Bacillus*, their abundance should increase with incubation, which was the opposite to what we found. We believe future studies on the eggshell microbes should investigate the interaction of microbes, because the incubation and/or environmental factors such as urbanization or climate condition can influence the dynamic interactions among the microbes on the eggshell.

Although we examined only one pair of urban-rural populations, the difference in breeding success that we detected is consistent with previous studies on magpies [80, 81]. Recently, the impact of urbanization on the clutch size and laying date of four species of small hole-nesting birds was thoroughly examined [82] and this study proposed many factors for the difference in birds’ breeding in the environment with varying degree of urbanization. Our results add another factor: microbial abundance that may influence hatching success and nestling survival. Whether the differences in eggshell microbial abundance between urban and rural populations of wild birds that we observed in this study can be generalized to other urban-rural habitat pairs or urban-rural gradients can be determined only after more rigorous studies are conducted. Future comparisons between urban and rural areas regarding microbial communities associated with wildlife should comprise detailed observations or comparisons in order to determine the differences in microbial sources and should also collect more samples from urban-rural gradients. In addition, for a better comparison between incubated and non-incubated eggs, one might consider blocking the entry of the parents in order to remove the effect of incubation while keeping the natural microbial community on the eggshells intact.

## Supporting information

S1 Table (XLSX)

S2 Table (DOCX)

S3 Table (DOCX)

S4 Table (DOCX)

S5 Table (DOCX)
