# Supplementary material for: Microbial abundance on the eggs of a passerine bird and related fitness consequences between urban and rural habitats
Source: PLoS One. 2017 Sep 27;12(9):e0185411. doi: 10.1371/journal.pone.0185411 (PMC5617198; doi:10.1371/journal.pone.0185411)
Supplement: S2 Table — (DOCX) [file pone.0185411.s002.docx]

Supporting Table 1. Comparisons between urban (n=17) and rural (n=14) magpie nests. The Chi-square (**χ^2^**) test was conducted from a 2x2 table, with the number of nests in two habitats and two types of trees.

| **Variable** | **Area** | **Mean** ± **SD** | **t or χ^2^** | **p** |
| --- | --- | --- | --- | --- |
| Clutch size | Urban | 6.00 ± 1.41 | t = 3.27 | 0.002 |
|  | Rural | 7.57 ± 1.22 |  |  |
| Laying date | Urban | 24.8 ± 6.2 | t = 0.13 | 0.268 |
|  | Rural | 22.6 ± 4.2 |  |  |
| Tree height | Urban | 11.4 ± 2.0 | t = 0.22 | 0.831 |
|  | Rural | 1.2 ± 3.0 |  |  |
| Nest height | Urban | 8.7 ± 1.8 | t = 1.22 | 0.235 |
|  | Rural | 7.8 ± 1.9 |  |  |
| Nest tree type (coniferous vs deciduous) | Urban | 1.9 ± 0.3 | χ^2^ = 2.52 | 0.112 |
|  | Rural | 1.6 ± 0.5 |  |  |
