# Supplementary material for: Microbial abundance on the eggs of a passerine bird and related fitness consequences between urban and rural habitats
Source: PLoS One. 2017 Sep 27;12(9):e0185411. doi: 10.1371/journal.pone.0185411 (PMC5617198; doi:10.1371/journal.pone.0185411)
Supplement: S3 Table — (DOCX) [file pone.0185411.s003.docx]

Supporting Table 2. The correlation between the microbial abundance values (n=31 nests). Statistical significance is noted as “*” for 0.01<p<0.05, “**” for 0.001<p<0.01 and “***” for p<0.0001.

|  |  | Day 3 abundance | | | | Day 18 abundance | | | |
| --- | --- | --- | --- | --- | --- | --- | --- | --- | --- |
|  |  | Total bacteria | *E. coli/Shigella* spp. | Surfactin-producing *Bacillus* spp. | *C. albicans* | Total bacteria | *E. coli/Shigella* spp. | Surfactin-producing *Bacillus* spp. | *C. albicans* |
| Day 3 abundance | Total bacteria | 1.000 | -0.234 | -0.139 | -0.261 | -0.273 | -0.113 | -0.376^*^ | -0.050 |
|  | *E. coli/Shigella* spp. |  | 1.000 | 0.608^***^ | 0.731^***^ | 0.035 | 0.322 | 0.459^**^ | 0.353 |
|  | Surfactin-producing *Bacillus* spp. |  |  | 1.000 | 0.369^*^ | -0.018 | 0.266 | 0.322 | 0.515^**^ |
|  | *C. albicans* |  |  |  | 1.000 | 0.075 | 0.481^**^ | 0.261 | 0.008 |
| Δ abundance | Total bacteria | -0.941^***^ | 0.210 | 0.111 | 0.247 |  |  |  |  |
|  | *E. coli/Shigella* spp. | -0.101 | 0.268 | 0.234 | 0.446^*^ |  |  |  |  |
|  | Surfactin-producing *Bacillus* spp. | -0.052 | -0.390^*^ | -0.869^***^ | -0.247 |  |  |  |  |
|  | *C. albicans* | 0.215 | -0.509^**^ | -0.112 | -0.902^***^ |  |  |  |  |
| Day 18 abundance | Total bacteria |  |  |  |  | 1.000 | -0.301 | 0.496^**^ | -0.351 |
|  | *E. coli/Shigella* spp. |  |  |  |  |  | 1.000 | 0.060 | 0.242 |
|  | Surfactin-producing *Bacillus* spp. |  |  |  |  |  |  | 1.000 | 0.237 |
|  | *C. albicans* |  |  |  |  |  |  |  | 1.000 |
