# Supplementary material for: Microbial abundance on the eggs of a passerine bird and related fitness consequences between urban and rural habitats
Source: PLoS One. 2017 Sep 27;12(9):e0185411. doi: 10.1371/journal.pone.0185411 (PMC5617198; doi:10.1371/journal.pone.0185411)
Supplement: S4 Table — (DOCX) [file pone.0185411.s004.docx]

Supporting Table 3. Composition of the principal components for microbial abundance.

1. For day 3 abundance

|  | PC1 | PC2 | PC3 |
| --- | --- | --- | --- |
| Total bacteria | -0.283 | 0.928 | 0.242 |
| *E. coli/Shigella* spp. | 0.610 | 0.163 | 0.138 |
| Surfactin-producing *Bacillus* spp. | 0.488 | 0.335 | -0.736 |
| *Candida albicans* | 0.556 | -0.001 | 0.617 |
| Eigenvalues | 2.260 | 0.909 | 0.621 |
| Proportion of variance | 0.565 | 0.227 | 0.155 |
| Cumulative proportion | 0.565 | 0.792 | 0.947 |

1. For day 18 abundance

|  | PC1 | PC2 | PC3 |
| --- | --- | --- | --- |
| Total bacteria | 0.704 | 0.200 | 0.173 |
| *Escherichia coli* | -0.455 | 0.344 | 0.803 |
| *Bacillus subtilis* | 0.341 | 0.934 | 0.002 |
| *Candida albicans* | -0.427 | 0.550 | -0.570 |
| Eigenvalues | 1.648 | 1.341 | 0.764 |
| Proportion of variance | 0.412 | 0.335 | 0.191 |
| Cumulative proportion | 0.412 | 0.747 | 0.938 |

1. For day 3 abundance and the difference in abundance between day 18 and day 3 (Δ)

| Microbial entity | Stage | PC1 | PC2 | PC3 |
| --- | --- | --- | --- | --- |
| Total bacteria | Day 3 | -0.257 | 0.527 | 0.360 |
|  | Δ | 0.236 | -0.567 | -0.337 |
| *E. coli/Shigella* spp. | Day 3 | 0.455 | 0.099 | 0.017 |
|  | Δ | 0.267 | 0.123 | 0.258 |
| Surfactin-producing *Bacillus* spp. | Day 3 | 0.368 | 0.353 | -0.436 |
|  | Δ | -0.276 | -0.480 | 0.377 |
| *Candida albicans* | Day 3 | 0.484 | -0.031 | 0.355 |
|  | Δ | -0.392 | 0.142 | -0.481 |
| Eigenvalues | | 3.346 | 1.973 | 1.366 |
| Proportion of variance | | 0.418 | 0.247 | 0.171 |
| Cumulative proportion | | 0.418 | 0.665 | 0.836 |
