# Supplementary material for: Microbial abundance on the eggs of a passerine bird and related fitness consequences between urban and rural habitats
Source: PLoS One. 2017 Sep 27;12(9):e0185411. doi: 10.1371/journal.pone.0185411 (PMC5617198; doi:10.1371/journal.pone.0185411)
Supplement: S5 Table — (DOCX) [file pone.0185411.s005.docx]

Supporting Table 4. Ecological factors for the microbial abundance (principal components). For (a) and (b), the numerical degree of freedom was 1 and the denominator degree of freedom was 27; for (c), they were 1 and 25 respectively.

1. Day 3 abundance

| Effect | | PC1 | | | PC2 | | | PC3 | | |
| --- | --- | --- | --- | --- | --- | --- | --- | --- | --- | --- |
|  |  | Estimate ±SE | F | P | Estimate ±SE | F | P | Estimate ±SE | F | P |
| Population | Rural | -5.522±0.452 | 9.76 | 0.004 |  | 2.83 | 0.104 |  | 0.01 | 0.934 |
|  | Urban | -4.110±2.505 |  |  |  |  |  |  |  |  |
| Av. Temperature (day 0-3) | | 0.396±0.132 | 9.02 | 0.006 |  | 0.00 | 0.963 | -0.186±0.080 | 5.38 | 0.028 |
| Av. Humidity (day 0-3) | |  | 1.18 | 0.288 |  | 1.69 | 0.205 |  | 1.52 | 0.209 |

1. Day 18 abundance

| Effect | PC1 | | | PC2 | | | PC3 | | |
| --- | --- | --- | --- | --- | --- | --- | --- | --- | --- |
|  | Estimate ±SE | F | P | Estimate ±SE | F | P | Estimate ±SE | F | P |
| Population |  | 0.04 | 0.838 |  | 0.17 | 0.684 |  | 2.50 | 0.125 |
| Av. Temperature (day 15-18) |  | 0.17 | 0.682 |  | 0.78 | 0.384 |  | 0.31 | 0.580 |
| Av. Humidity (day 15-18) |  | 0.07 | 0.788 | 0.090±0.040 | 5.09 | 0.032 | -0.055±0.031 | 3.12 | 0.089 |

1. Day 3 abundance and difference

| Effect | | PC1 | | | PC2 | | | PC3 | | |
| --- | --- | --- | --- | --- | --- | --- | --- | --- | --- | --- |
|  |  | Estimate ±SE | F | P | Estimate ±SE | F | P | Estimate ±SE | F | P |
| Population | Rural | -3.112±0.671 | 4.49 | 0.041 |  | 1.95 | 0.175 |  | 0.03 | 0.854 |
|  | Urban | -1.689±5.631 |  |  |  |  |  |  |  |  |
| Av. Temperature (day 0-3) | | 0.326±0.181 | 3.25 | 0.084 | 0.256±0.089 | 8.18 | 0.008 | -0.256±0.120 | 4.52 | 0.044 |
| Av. Humidity (day 0-3) | |  | 0.48 | 0.493 |  | 1.90 | 0.181 |  | 0.46 | 0.505 |
| Temperature difference | |  | 1.54 | 0.226 | -1.010±0.134 | 57.19 | 0.000 |  | 3.05 | 0.093 |
| Av. Humidity (day 15-18) | |  | 0.24 | 0.626 |  | 0.47 | 0.497 |  | 0.00 | 0.964 |
